# Under-Recognized Life-Threatening Vasovagal Reflex During Chest Tube Insertion

**DOI:** 10.7759/cureus.61226

**Published:** 2024-05-28

**Authors:** Yuji Okazaki, Kyungko Huh, Toshihisa Ichiba

**Affiliations:** 1 Emergency Medicine, Hiroshima City Hiroshima Citizens Hospital, Hiroshima, JPN

**Keywords:** vasovagal reflex, pneumothorax, chest tube insertion, cardiac arrest, atropine

## Abstract

Chest tube insertion is a common and relatively safe procedure in an emergency setting. However, a potentially fatal complication, vasovagal reflex, may be under-recognized due to its generally mild severity. We present a case of pulseless electrical activity (PEA) requiring chest compression due to vasovagal reflex during chest tube insertion for spontaneous pneumothorax. A 23-year-old male who had a history of spontaneous pneumothorax presented with left chest pain to our emergency department. Based on point-of-care ultrasonography and chest radiography, we made a diagnosis of recurrent pneumothorax. Although he had stable vital signs and received adequate pain control, during chest tube insertion, he developed severe sinus bradycardia with a six-second pause, leading to PEA requiring chest compressions. After a few compressions, his heart rate increased and he regained consciousness. He underwent video-assisted thoracoscopic surgery for pneumothorax and was discharged without complications. Vasovagal reflex during chest tube insertion in young patients with spontaneous pneumothorax may cause severe bradycardia and cardiac arrest. Physicians should be aware of this rare but potentially fatal complication and be prepared with appropriate measures, such as pre-administration of atropine, before chest tube insertion.

## Introduction

Insertion of a chest tube for pneumothorax is a common procedure performed in an emergency setting. This is generally a painful procedure, necessitating adequate analgesia and mild sedation to decrease discomfort [1,2]. When performing the insertion, while careful attention is paid to serious complications including injury of arteries and visceral organs, vasovagal reflex, which is occasionally experienced, is often not considered a significant concern for physicians. Although the exact frequency of this reflex during chest tube insertion is unknown, it has been reported to occur in 2.4% of cases [3]. In addition, even if it occurs, the reflex usually improves with conservative treatment. However, this may unexpectedly cause severe bradycardia. Delays in recognizing and responding to this complication can lead to cardiac arrest. We present a case of pulseless electrical activity (PEA) requiring chest compression due to vasovagal reflex during chest tube insertion for spontaneous pneumothorax in a young male.

## Case presentation

**Figure 1:**
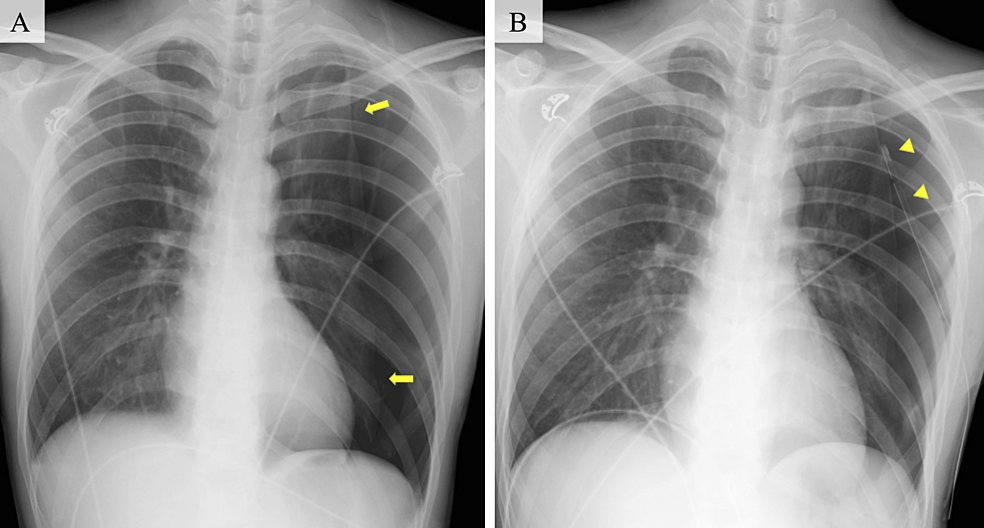
Chest radiography (A) An AP chest radiograph showing a collapsed left lung without pleural effusion (yellow arrow). (B) An AP chest radiograph showing an inserted chest tube in the left thorax (yellow arrowhead). AP: anterior-posterior

A 23-year-old male presented to our emergency department with a complaint of left chest pain two hours after onset. He had undergone chest tube insertion and video-assisted thoracoscopic bullectomy for left spontaneous pneumothorax two weeks before. He had not experienced vasovagal syncope. On arrival, his vital signs were as follows: body temperature, 36°; blood pressure, 163/74 mmHg; heart rate, 91 beats per minute; respiratory rate, 34 per minute; and oxygen saturation, 96% without supplementation of oxygen. Physical examination revealed decreased breath sounds in the left lung. Point-of-care ultrasonography showed absence of the lung sliding sign on the left side of the chest, and chest radiography showed a collapsed left lung without pleural effusion (Figure 1A), leading to a diagnosis of recurrent pneumothorax.

**Figure 2:**
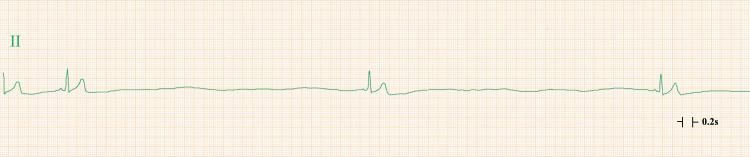
Electrocardiogram monitoring Electrocardiogram monitoring showing a six-second pause with P waves and narrow QRS complexes, consistent with severe sinus bradycardia.

Acetaminophen (1,000 mg) was administered intravenously, and 10 mL of 1% lidocaine was administered subcutaneously for local anesthesia. During the dissection of subcutaneous tissue around the ribs in preparation for the insertion of a 12 Fr chest tube, he complained of mild left chest pain and developed severe sinus bradycardia. Atropine (0.5 mg) was administered intravenously; however, the bradycardia persisted and was accompanied by a six-second pause, consistent with severe sinus bradycardia (Figure 2).

He became unconscious, and physicians performed chest compressions based on the judgment of PEA. After a few chest compressions, his heart rate increased and his consciousness recovered. His vital signs were as follows: blood pressure, 114/55 mmHg; heart rate, 114 beats per minute; respiratory rate, 42 per minute; and oxygen saturation, 100% on 10 L per minute of supplemental oxygen. After regaining consciousness, he had no specific complaints. A 12-lead electrocardiogram (ECG) showed P waves with narrow QRS complexes at a heart rate of 105 beats per minute and no changes in the ST segments or T waves. Laboratory examinations also showed no abnormalities. Therefore, we made a diagnosis of cardiac arrest due to vasovagal reflex. A chest tube was successfully inserted (Figure 1B). He underwent video-assisted thoracoscopic surgery for the pneumothorax on day 2 of admission and was discharged on day 6 without any complications.

## Discussion

Vasovagal reflex during chest tube insertion in young patients with spontaneous pneumothorax can cause severe bradycardia and lead to cardiac arrest. During chest tube insertion, even if this reflex occurs, it is usually mild and self-limiting. Thus, its potential to cause severe bradycardia may be under-recognized. However, there are two reasons for the development of the potentially fatal complication during chest tube insertion in patients with spontaneous pneumothorax. First, vasovagal reflex is elicited by emotional stress due to pain or fear of bodily injury, and it is more likely to occur in young individuals than in the elderly [4,5]. Young people who have spontaneous pneumothorax are usually healthy, and although chest tube insertion was not the first experience in our case, it is likely to be unfamiliar with such medical procedures. Therefore, in young patients, the emotional stress and painful procedure may cause a stronger vasovagal reflex. Second, stimulation of the vagus nerve may occur during the placement of a chest tube, leading to severe bradycardia. This may be due to direct stimulation of branches of the vagus nerve after the insertion of the chest tube into the thorax [6]. In addition, a case of sudden death due to vagal stimulation after chest tube insertion in a patient with traumatic hemothorax has been reported [7]. In other words, strong stimulation of the parasympathetic nervous system, whether by a tube or a hematoma, may cause cardiac arrest. Although these factors may combine to cause severe vasovagal reflex, our patient had PEA before the insertion of the tube into the thoracic cavity, indicating that caution should be exercised even before penetrating the pleura.

Considering hemodynamic collapse due to vasovagal reflex during chest insertion, we cannot ignore the impact of pneumothorax on hemodynamic status. Patients with pneumothorax, whether tension pneumothorax or not, have increased intrathoracic pressure, leading to a decrease in venous return [8]. In addition, in the case of hemopneumothorax, the decrease in intravascular volume would further lower the preload. Under these conditions, vasovagal reflex may be more likely to cause hemodynamic compromise.

## Conclusions

Physicians should be well aware of severe bradycardia due to vasovagal reflex as a life-threatening complication during chest tube insertion. Considering that chest tube insertion is a frequently performed procedure in an emergency department, although this complication is rare, it is important to be fully prepared for this potentially fatal complication and take appropriate precautions, such as pre-administration of atropine, before inserting a chest tube. If bradycardia occurs during the procedure, prompt intervention is recommended rather than immediately assuming it is transient. Further reporting is warranted to better recognize this complication in the emergency community.
